# Antidiarrheal Potential of *Viola canescens*: In Vivo and In Silico Approaches

**DOI:** 10.3390/ph16040489

**Published:** 2023-03-25

**Authors:** Imtiaz Ahmad, Bader S. Alotaibi, Nosheen Malak, Fayaz Asad, Barkat Ullah, Nasreen Nasreen, Adil Khan, Chien-Chin Chen

**Affiliations:** 1 Department of Botany and Zoology, Bacha Khan University Charsadda, Charsadda 24420, Pakistan; 2 Department of Botany, University of Peshawar, Peshawar 25120, Pakistan; 3 Departmet of Laboratories Sciences, College of Applied Medical Sciences, Al Quwayiyah, Shaqra University, Shaqra 13253, Saudi Arabia; 4 Department of Zoology, Abdul Wali Khan University Mardan, Mardan 23200, Pakistan; 5 Department of Botany, Islamia College University Peshawar, Peshawar 25120, Pakistan; 6 Department of Pathology, Ditmanson Medical Foundation Chia-Yi Christian Hospital, Chiayi 600566, Taiwan; 7 Department of Cosmetic Science, Chia Nan University of Pharmacy and Science, Tainan 71710, Taiwan; 8 Department of Biotechnology and Bioindustry Sciences, National Cheng Kung University, Tainan 70101, Taiwan; 9 Ph.D. Program in Translational Medicine, Rong Hsing Research Center for Translational Medicine, National Chung Hsing University, Taichung 40227, Taiwan

**Keywords:** antidiarrheal, *Viola canescens*, quercetin, violanthin, charcoal assay, molecular docking, phytocompounds

## Abstract

*Viola canescens* Wall. is an important medicinal plant with reported therapeutic benefits. The current work sought to investigate the antidiarrheal properties of *V. canescens* extracts both in vivo and in silico. This study applied molecular docking to unravel the molecular mechanism of *V. canescens* and to find the most effective phytocompounds with antidiarrheal effects. The antidiarrheal activity of *V. canescens* was assessed utilizing the castor oil-induced diarrhea assay and the charcoal meal assay. Antidiarrheal characteristics were evaluated by measuring parameters such as intestinal motility, fecal score, and hypersecretion. The *V. canescens* extract had a dose-dependent and statistically significant impact in the charcoal meal assay and castor oil-induced diarrhea assay. In the castor oil-induced diarrhea assay, the ethyl acetate fraction (65.96%) showed the highest percentage of defecation inhibition at the highest dose (300 mg/kg (bw)), followed by the uncorrected crystalline compound (63.83%), crude alkaloids (63.83%), chloroform fraction (63.83%), and crude flavonoids (55.32%), while the aqueous fraction (40.43%) and n-Hexane fraction (42.55%) revealed the lowest antidiarrheal potential. In addition, the molecular docking investigation showed emetine, quercetin, and violanthin, isolated chemicals of *V. canescens*, to have the highest binding affinity to the target μ and δ opioid receptors with significant inhibitory capacity. These pharmacologically active metabolites in *V. canescens* were effective in treating diarrhea. This study lends credence to the traditional usage of *V. canescens* in treating gastrointestinal disorders.

## 1. Introduction

Diarrhea is a medical condition caused by pathogenic bacteria, viruses, and other pathogens, indicated by frequent wet fecal output, increased concentration of solutes in the intestinal cavity, excessive release of electrolytes, the inability of the intestine to absorb optimum electrolytes, and increased intestinal motility. Major diarrhea-causing bacteria include *Shigella flexneri*, *Salmonella typhi*, and *E. coli*. The fungus *Candida albicans* has also been identified as a major causal agent [1]. Diarrhea occurs when the secretion and absorption capacity of the small intestine become upset by bacterial or other pathogenic infections. In severe conditions, especially in infants and older adults, diarrhea can be lethal because of excessive water loss and electrolytes from the body. The frequency of diarrheal infection and mortality is high in underdeveloped countries with low hygiene, and approximately 525,000 deaths occur because of diarrheal infection worldwide [2,3]. Diarrheal infections can aid vulnerability to other infections by adversely affecting the immune system, which may be a possible factor for the high mortality rate in individuals with secondary infections [4]. Considering the adverse side effects of synthetic drugs and the significant decrease in the mortality rate related to using plant-derived substances, the search for potential plant products has gained immense importance [5].

*Viola canescens* Wall. belongs to the family Violaceae, a small herb (10–25 cm tall) with immense ethnomedicinal importance. The plant bears cylindrical and branched roots. The stem is absent. Leaves are basal, broadly ovate, or cordate with dimensions of 2.0–10 × 1.0–5.0 cm. The petiole is longer than the lamina. Flowers are solitary, violet to pale violet in color. The plant is distributed among temperate and tropical zones, mainly confined to mountainous areas. In Pakistan, the plant species have been reported from various localities, including Kashmir, Poonch, Abbottabad, Balakot, Swat, and Murree. Globally *V. canescens* has been reported in India, Nepal, and Bhutan [6].

*V. canescens* is a rich source of secondary metabolites and contains methyl salicylate, violin, and emetine (alkaloids), quercetin, luteolin, and rutin (flavonoids), epicatechin, vanillic acid, p-coumaric acid, ferulic acid, syringic acid, and caffeic acid (phenolic acids), saponins, and glucosides [7,8,9]. In traditional healthcare systems, the plant is an antipyretic, anti-inflammatory, and antispasmodic agent for several therapeutic purposes, including relieving digestive problems [6,7]. The plant is also reported to possess antimicrobial, antispasmodic, and hepatoprotective potentials and other properties such as antioxidant, analgesic, diaphoretic, carminative, and aphrodisiac effects [8,9]. Nevertheless, no significant work has previously been conducted on in vivo and in silico antidiarrheal activities of the plant, therefore based on the use of the plant for relieving digestive disorders in traditional care systems [10] and the significant inhibitory potential of the plant against *E. coli* and *C. albicans*, the major diarrhea-inducing organisms [6,11], the present study aimed to evaluate the antidiarrheal potential and to identify the potential antidiarrheal constituents of *V. canescens* using in silico and in vivo approaches.

## 2. Results

### 2.1. Castor Oil-Induced Diarrhea Assay

Crude methanolic extract (CME) and all other fractions exhibited significant (*p* < 0.01) antidiarrheal activities at selected doses (100 mg/kg, 200 mg/kg, and 300 mg/kg body weight (bw)). In this model, the antidiarrheal potential of a plant (extracts/fractions) was assessed by calculating the % inhibition of defecation and % fecal output. Significant inhibition in the frequency of defection, compared to the negative control, was recorded for all treatments. The % fecal output was also significantly reduced by all fractions at selected doses. A strong positive correlation was observed between the antidiarrheal potential of treatment and dose concentration. Maximum % inhibition of defecation at the highest dose (300 mg/kg (bw)) was recorded for ethyl acetate fraction (EAF) (65.96%), followed by uncharacterized crystalline compound (UCC) (63.83%), crude alkaloids (CTA) (63.83%), chloroform fraction (ChF) (63.83%), and crude flavonoids (CTF) (55.32%), while the least activity was observed for the aqueous fraction (AqF) (40.43%) and n-Hexane fraction (NHF) (42.55%). Maximum reduction in % fecal output was recorded for CTF (53.19%) followed by UCC (52.68%), CTA (48.375), and EAF (45.97). CME and all other fractions significantly delayed the onset of diarrheal symptoms at selected doses in a dose-dependent manner. The highest dose (300 mg/kg (bw)), maximum delay (184.0 ± 2.24 min) was caused by CTA followed by UCC (171.8 ± 164 min) and ChF (149.4 ± 2.19 min). The number of wet feces and weight of wet feces were also significantly decreased by CME and other fractions at selected doses. EAF, CTA, and CME were found most effective in this regard, with no. of wet feces recorded as 3.2 ± 0.45, 3.6 ± 0.55, and 3.4 ± 0.55, respectively, while the weight of wet feces as 2.74 ± 0.05 g, 2.92 ± 0.13 g, and 2.97 ± 0.30 g, respectively. In general, all extracts/fractions exhibited significant antidiarrheal activities comparable with the activities of loperamide (Table 1).

### 2.2. Charcoal Meal (Gastro Motility) Assay

All extracts/fractions at selected doses significantly (*p* < 0.01) reduced the movement of charcoal meal in the small intestine. Maximum activities for all extracts/fractions were recorded at the highest concentration. The highest activity was recorded for CTF, ChF, CTA, and UCC, with % inhibition values of 58.08, 52.51, 48.52, and 46.70, respectively. The least inhibition was recorded for AqF and NHF, with a % inhibition of 29.74% and 30.87%, respectively. The mean distance covered by charcoal in the small intestine was significantly increased (*p* < 0.01) by CME and all other fractions in a dose-dependent manner. Maximum decrease at the highest concentration (300 mg/kg (bw)) was recorded for ChF (23.04 ± 1.26), followed by CTA (24.98 ± 1.65) and UCC (25.98 ± 1.02). N-Hexane was found to be the least effective treatment (33.54 ± 1.10). A significant decrease in the peristalsis index was also recorded for CME and all other fractions compared to normal saline (negative control). Maximum activities at the highest concentration (300 mg/kg (bw)) were recorded for ChF, CTA, and EAF with a peristalsis index of 48.10, 49.7, and 55.78, respectively. The activities of these treatments are comparable with atropine sulfate used as the positive control (Table 2).

### 2.3. Docking Studies

**Figure 1:**
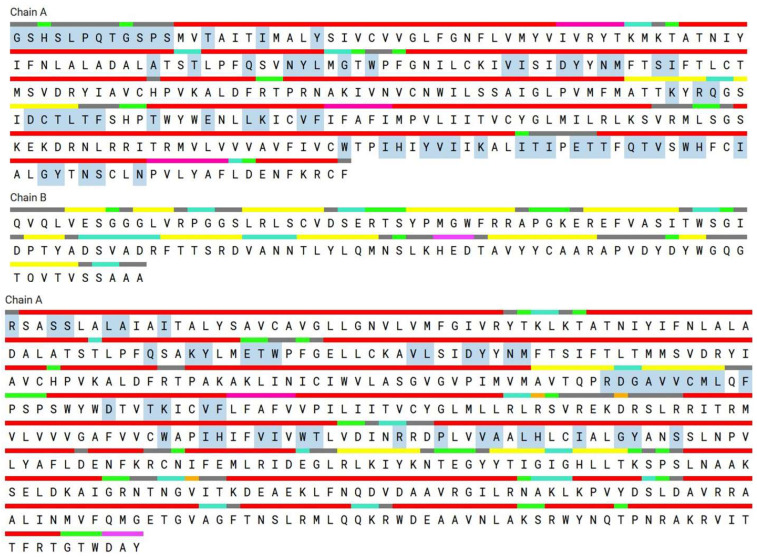
Active sites of 5C1M and 4EJ4 protein found through CASTp server.

After preparing the chemicals and enzymes, AutoDock Vina software was used for docking [12]. The active site residues were determined to be the amino acid residues interacting with the co-crystallized ligands of both the 5CIM and 4EJ4, and docking grid parameters were established accordingly. They are MET132, TYR129, ASP128, THR213, LYS214, VAL217, PHE218, TRP274, ILE277, HIS278, ILE277, and TYR308 for 4EJ4 protein and SER53, HIS54, SER55, TYR75, PHE123, GLN124, SER125, ASN127, TYR128, TRP133, ASP147, TYR148, MET151, LEU232, LYS233, ILE234, CYS235, VAL236, PHE237, TRP293, ILE296, HIS297, VAL300, LYS303, TRP318, HIS319, ILE322, and TYR236 amino acids for 5CIM protein (Figure 1).

The location of the ligand inside the protein-binding site was mapped using Autogrid. The dimensions (angstrom) for the 5CIM protein were determined to be X: 24.0000 Å, Y: 32.0001 Å, and Z: 31.0214 Å, and for the 4EJ4 protein, they were determined to be X: 64.0289 Å, Y: 41.9033 Å, and Z: −9.80663 Å. This investigation thus resolved a center coordinate of X: 2.0000 Å, Y: 21.1256 Å, and Z: −64.2055 Å for 5CIM and X: 24.0000 Å, Y: 24.0000 Å, and Z: 24.0000 Å for 4EJ4 protein. The docking procedure was run with the standard exhaustiveness setting of 8. The postures with the lowest binding energy (kcal/mol) were chosen from the docking results for 5CIM and 4EJ4 and then visualized using Discovery Studio.

**Figure 2:**
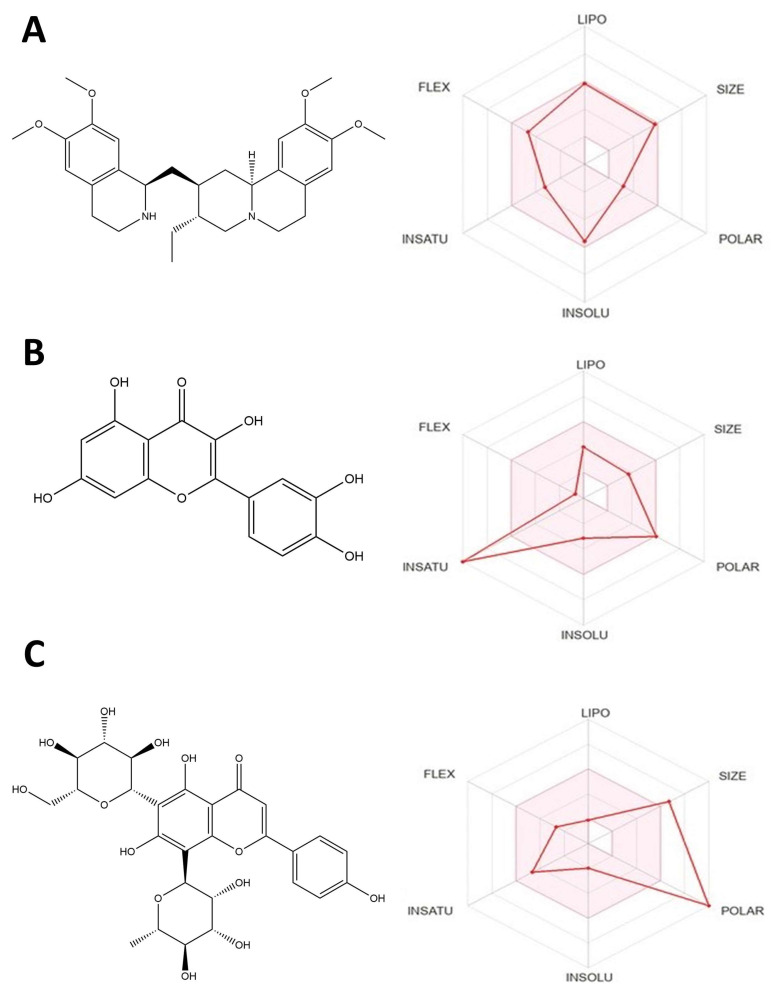
(**A**) ADMET bars for emetine (compound **1**). (**B**) ADMET bars for quercetin (compound **2**). (**C**) ADMET bars for violanthin (compound **3**).

**Figure 3:**
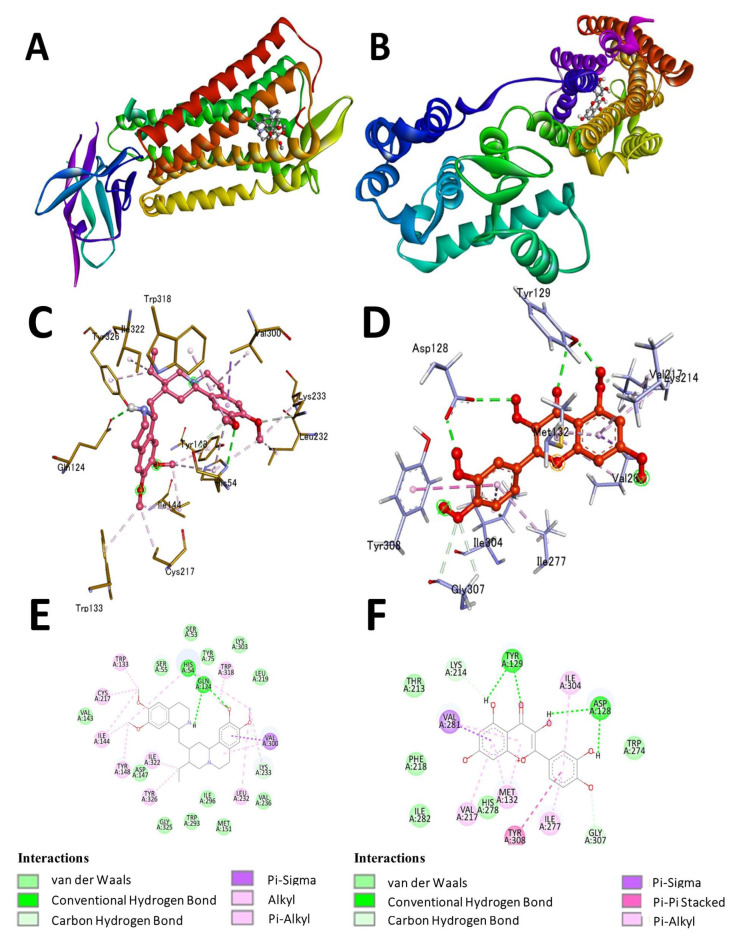
(**A**) 5C1M protein, (**B**) 4EJ4 protein, (**C**) the 3D interaction of emetine with 5C1M protein, (**D**) the 3D interaction of quercetin with 4EJ4 protein, (**E**) the 2D interaction of emetine with 5C1M protein, and (**F**) the 2D interaction of quercetin with 4EJ4 protein.

In order to promote comprehension of potential mechanisms of biological activity, docking simulations were conducted. AutoDock Vina analyzed the compounds’ docking behavior with *Mus musculus* opioid receptors (ORs), including muOR (μOR, PDB ID: 5C1M) and deltaOR (δOR, PDB ID: 4EJ4). The binding energy is negative for the examined inhibitors. The binding energy of compound **1** (emetine) (Figure 2A) to the 5C1M receptor is −9.7 kcal/mol, whereas that of compound **1** to the 4EJ4 is −7.1 kcal/mol, demonstrating significant inhibitory capacity. Compound **2** (quercetin) (Figure 2B) shows the best binding energy of −8.5 kcal/mol against the δOR 4EJ4 receptor, while −8.1 kcal/mol against the μOR 5C1M receptor. Compound **3** (violanthin) (Figure 2C) shows high binding energy of −9.5 kcal/mol against the 5C1M receptor and −7.9 kcal/mol against the 4EJ4 receptor. The stability in ligand binding is greatly aided by hydrogen bonding, and the maximum acceptable bond gap between H acceptor and H donor atoms is 3.5 Å [13]. Two hydrogen bonding interactions exist between compound **1** and the 5C1M receptor (Figure 3A). Bond lengths of 1.49 and 1.46 indicate a hydrogen connection between the His54 and Gln124 amino acid residues. Hydrophobic contacts were made between the amino acid residues Trp133, Cys217, Ile144, Tyr148, Ile322, Tyr326, Leu232, Lys233, Val300, and Trp318 (Figure 3C,E). Asp128 and Tyr129 form a double hydrogen bond in compound **2**, whereas Met132, Val217, Val281, Ile277, Gly307, Tyr308, and Lys214 form hydrophobic contacts in the 4EJ4 receptor (Figure 3B,D,E). The results showed compound **2** (quercetin) had the most potent inhibitory effect against the δOR 4EJ4 receptor, whereas compound **1** (emetine) had the greatest ability to inhibit the μOR 5C1M receptor. The findings are presented in Table 3.

### 2.4. Molecular Dynamics (MD) Simulation

**Figure 4:**
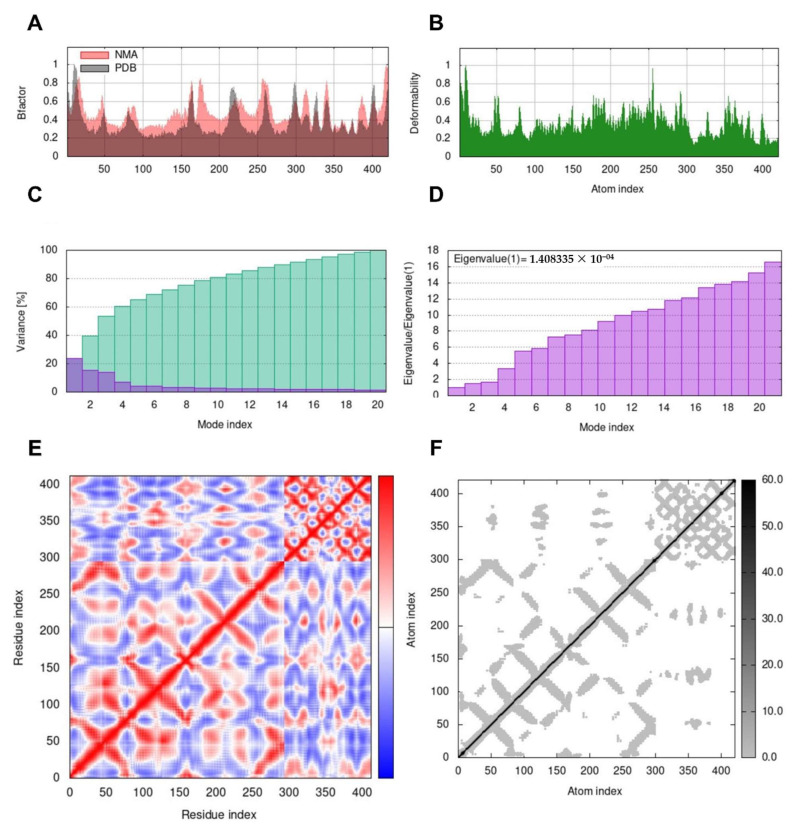
Molecular dynamics’ simulation of the predicted ligand–protein complex; complex stability was assessed through (**A**) deformability, (**B**) B-factor values, (**C**) variance, (**D**) eigenvalue, (**E**) the covariance of residue index, and (**F**) elastic network analysis.

MD simulation was used to verify the docking studies and assess the modification in the protein structure. MD was simulated using the iMODS server to examine the ligand–receptor combination’s stability. The chain’s hinges represent each residue’s distortion to determine the complex’s deformability (Figure 4B). Thus, 1.41 × 10^−4^ was discovered to be the complex’s estimated eigenvalue (Figure 4D). In general, each normal mode’s eigenvalue and associated variance were determined to be inversely connected (Figure 4C). The iMODS server’s B-factor scores for normal mode analysis were equal to RMS scores (Figure 4A). Different pairings of residues showed correlated, anti-correlated, or uncorrelated motions, represented by the colors red, blue, and white in the covariance matrix, which described the connection between pairs of residues (Figure 4E). Finally, the server created an elastic network model (Figure 4F), which displayed pairs of atoms connected by springs in accordance with their degree of stiffness. This was depicted by color, moving from lighter grey with softer strings to a darker grey with stiffer strings.

### 2.5. Drug Likeness and Pharmacokinetics Properties

The ADMET properties of the top-performing drug and common inhibitors were predicted in this study using the Swiss ADME and pkCSM web servers. Tables were used to present the results of the ADMET test. We discovered from the findings that some chemicals broke Lipinski’s rule of five (RO5). For instance, violanthin broke three of Lipinski’s rules of five, although chemicals such as emetine and quercetin did not (Table 4). We also looked at the physiochemical parameters gleaned from the pkCSM web server, in addition to the physicochemical and drug-likeness properties taken into account in Lipinski’s RO5.

A chemical is deemed to have a high Caco-2 permeability for the pkCSM webserver if its log Papp value is more than 0.90 cm/s [9]. Table 5 shows that all substances will have poor intestinal absorption when they have Caco-2 and intestinal absorbance values of 0.90 and 30%, respectively. It is suggested that a medication’s skin permeability (log Kp), a crucial factor in enhancing therapeutic efficacy and particularly relevant in transdermal drug administration development, be set at more than −2.5 cm/h [8]. All compounds have log Kp values ranging from −2.8 to −3.6 cm/h. All substances are therefore expected to have good skin penetration. The blood–brain barrier (BBB) and the volume of distribution at steady state (VDss) are crucial factors to consider when assessing a drug’s capacity to cross the blood–brain barrier. The amount of medicine delivered to tissue instead of plasma increases with increasing VD. The model was created using the steady-state volume of distribution (VDss) estimations. According to Pires et al. (2015), a chemical has an excellent distribution if its VDss value is greater than 0.45. Almost all compounds have VDss values that are twice as high as recommended. A molecule can quickly pass through the BBB when log BB is higher than 0.3, which is the BBB’s definition of a medicine’s ability to enter the brain while boosting drug efficacy (fewer adverse effects). All drugs under examination have a moderate ability to pass the blood–brain barrier because their log BB values are lower than 0.3.

According to the studies, only CYP2D6 and CYP3A4 are involved in the metabolism of drugs. The biotransformation, metabolism, and/or detoxification of xenobiotics in the body, are made possible by specialized enzymes, including cytochromes P450. In terms of metabolism, we discovered that only emetine is likely to be metabolized by CYP2D6 and CYP3A4. A medicine’s total clearance (TC) number represents its capacity to be eliminated by many organs, such as the liver and the kidneys. The TC for each chemical was provided in this study. Our research shows emetine has the highest clearance value, 0.99 logs (mL/min/kg). To conclude categorically with predictions of toxicities, key indicators for toxicity include oral rat acute toxicity LD_50_ and, therefore, toxicity class, as well as mutagenicity and hepatotoxicity to some extent.

The toxicity results show that none of the chemicals were mutagenic or hepatotoxic. Their LD_50_ values, however, range from 2.5 to 2.73 mol/kg.

## 3. Discussion

The best approach to treat diarrheal symptoms is to use agents that help to reduce intestinal motility and retard the excessive release of electrolytes. In the castor oil-induced diarrheal model, diarrhea is caused by ricinoleic acid (an active constituent of castor oil) promoting gastrointestinal motility and release of electrolytes by triggering prostaglandin production [14]. Therefore, the antidiarrheal potential of extracts/fractions in this model may be associated with the inhibition of prostaglandin and, thus, decreasing the intestinal motility and secretion of electrolytes.

Extracts/fractions of *V. canescens* significantly reduced the rate of defection and % fecal output (castor oil-induced diarrhea model) and the movement of charcoal meal in the small intestine (charcoal meal assay), which may be associated with the potential of extracts/fractions to inhibit the production of prostaglandin. Several alkaloids, flavonoids, and other secondary metabolites, including quercetin, emetine, and violanthin, have been isolated from *V. canescens* [15], which are most probably responsible for the demonstrated antidiarrheal potential of the plant. Docking studies also confirmed the antidiarrheal potential of quercetin, emetine, and violanthin.

The most popular and often applied methods for analyzing the interaction between ligand–protein complexes at the atomic level are molecular docking and MD simulation. These methods can be used to create brand-new inhibitors that target biological targets that cause disease [16,17]. In the current work, three phytocompounds were screened from the *V. canescens* plant’s μOR and δOR antidiarrheal inhibitory properties. Diarrhea is reduced when mu- and delta-opioid receptors are targeted [18,19]. In order to identify prospective antidiarrheal inhibitors, specifically against μOR and δOR enzymes, a total of three phytochemicals were chosen and virtually screened by molecular docking, MD, and drug-likeness/ADMET assays.

The protein–ligand complex generated with low energy conformation is examined for the most advantageous docked pose-binding mode. Docking determines the ligands’ optimal binding orientation for the associated target molecules. Docking aids in evaluating the biological effects of small compounds by predicting binding affinity against the target protein [20]. During the docking investigation, the AutoDock Vina program docked all the phytochemicals into the two distinct protein molecules’ anticipated active sites. Compounds having well-defined chemical interactions with several active site residues demonstrated a predictable binding affinity against target protein molecules (μOR and δOR). The flexibility and stability of the complexes can be evaluated by MD simulation. Standard mode analysis was performed, built into the iMODS server, and the output data were analyzed, which characterized the collective functional motions of the complex, to obtain a deeper look at the docked complex.

All of these compounds have sufficient drug-like properties, according to the results of the drug-likeness test. Two compounds followed the Veber rule [21] and Lipinski’s rule of five [22]. According to ADMET prediction, their satisfactory Log P attribute is likely to cause their good intestine absorption. The failure of most drug candidates in the late phase of drug discovery is attributed to their unsatisfactory ADMET profiles [23]. In addition, lower levels of medication absorption and oral bioavailability are attributable to poor solubility and insufficient intestinal absorption [24].

Through docking and MD modeling experiments, three phytochemicals were discovered to have exceptional antidiarrheal inhibitory properties (best-hit compounds), especially against μOR and δOR proteins. These substances were violanthin, quercetin, and emetine. The plant *V. canescens* contains these compounds. A majority of detected substances are flavones, flavonoids, and/or alkaloids, which are naturally abundant and widely used in traditional Chinese and/or Ayurvedic medicines. The interaction between emetine and the μOR protein is considerably more substantial than the interactions between emetine, quercetin, violanthin, and δOR. It becomes clear from a rigorous review of the MD data that other non-bonding interactions, such as carbon–hydrogen bonds and hydrophobic contact, are subordinate to conventional hydrogen bonding. The anticipated catalytic/active sites of protein molecules are where amino acid residues are involved in different interactions. Emetine’s interaction with the μOR protein is far more promising than emetine’s interaction with δOR.

## 4. Materials and Methods

### 4.1. Collection of Plant Materials

Whole plants of *V. canescens* were collected at the flowering stage from April to June from different localities of District Swat (34^0^ 56/23.00//N and 72^0^ 14/19.41//E with an elevation of 6147 feet) and Dir (34^0^ 46/23.24//N and 71^0^ 57/20.59//E with an elevation of 4828 feet). Plant identification was performed using the standard literature [25] and further confirmed through the Department of Botany, University of Peshawar. The identified specimen was kept at the Herbarium, University of Peshawar, and recorded with accession No. Bot.20220 (PUP).

### 4.2. Preparation of Extracts and Fractions

Plant material (whole plant) was soaked in methanol at the rate of 100 gm of powder per 250 mL of solvent (methanol) in a closed container for seven days. During the soaking time, the mixture was vigorously shaken at regular intervals. The mixture was then filtered using Whatman filter paper No. 1. The filtrate was dried using a rotary evaporator to obtain CME. The extraction was conducted in triplicates. Organic solvent fractions NHF, EAF, ChF, and AqF of methanolic extracts were prepared using separating funnels. Furthermore, standard protocols separated CTA and CTF from methanolic extracts. Each fraction was prepared in triplicate. A UCC was also separated using column chromatography.

### 4.3. In Vivo Assays

#### 4.3.1. Ethical Committee Approval

The current study comprising in vivo studies on mice models was approved by the Ethical Committee of the Biological Sciences Section, Department of Botany, University of Peshawar, Pakistan, under approval no. 99-J-10849.

#### 4.3.2. Experimental Animals

Swiss albino mice of both sexes were obtained from the National Institutes of Health, Islamabad, Pakistan, and guidelines published in the “Guide for the care and use of laboratory animals” were strictly followed during the animal procedures.

#### 4.3.3. Castor Oil-Induced Diarrhea Assay

The standard protocol proposed by Awouters, et al. [26] was followed for the castor oil diarrhea assay. Swiss albino mice of both sexes (6–7 weeks of age) weighing 25–30 g were divided into 26 groups, each with five animals. The animals were kept on fasting for 18 h with free access to water before the commencement of treatment. To induce diarrhea in animals, a dose of 10 mL/kg body weight of castor oil was orally administered to all groups. After 30 min, normal saline (10 mL/kg) and loperamide (5 mg/kg) were given orally to groups I and II, designated as the negative and the positive control, respectively. The remaining 24 groups were administered orally with selected doses (100 mg, 200 mg, and 300 mg/kg body weight) of CME, EAF, AqF, ChF, NHF, UCC, CTA, and CTF. Animals of each group were kept in separate transparent chambers with bases lined with filter papers. The filter paper for each group (chamber) was changed for four consecutive hours. The time of onset of diarrhea, total no. of feces, total no. of wet feces, the total weight of feces, and the total weight of wet feces were recorded for each group. Percent antidiarrheal activity (% inhibition) and fecal output were calculated for each group using the following formula.

$$\%Activity=\frac{Mean\;No.\;of\;wet\;feces\;in\;control\;-\;Mean\;no.\;of\;feces\;in\;treatment}{Mean\;No.\;of\;wet\;feces\;in\;control}\times 100$$

$$\%wet\;fecal\;output=\frac{mean\;weigt\;of\;wet\;feces\;in\;treatment}{mean\;wight\;of\;wet\;feces\;in\;control}\times 100$$

#### 4.3.4. Charcoal Meal Assay

A charcoal meal assay was used to evaluate the effects of *V. canescens* on gastrointestinal movement. Swiss albino mice of both sexes (6–7 weeks of age) weighing 25–30 g were divided into 26 groups, each with five animals. Group I, selected as the negative control, received normal saline (orally) at the rate of 10 mL/kg body weight. Group II, selected as the positive control, received atropine sulfate 5 mg/kg body weight (ip). The remaining 24 groups were administered orally with selected doses (100 mg, 200 mg, and 300 mg/kg body weight) of CME, EAF, AqF, ChF, NHF, UCC, CTA, and CTF. After 30 min of dose administration, animals of each group were given 2 mL charcoal meal (activated charcoal suspension) orally. After 30 min of charcoal meal feeding, the animals were sacrificed through the procedure of cervical dislocation.

The small intestine of each animal was separated, and the total length of the small intestine and the distance traveled by charcoal meal were recorded for each animal. Percent inhibition and peristalsis index were calculated for each group using the following formula [27,28,29].

$$Percent\;inhibiton=\frac{S\;traveled\;by\;CM\;in\;control\;-\;S\;traveled\;by\;CM\;in\;treatment}{S\;traveled\;by\;CM\;in\;control}\times 100$$

$$Peristalsis\;index=\frac{Mean\;S\;traveled\;by\;CM\;in\;small\;intestine}{total\;lenght\;ofsmall\;intestine}\times 100$$

where “*S*” means distance and “*CM*” means charcoal meal

### 4.4. Docking and Simulation

#### 4.4.1. Selection and Preparation of Phytochemicals

The known phytochemicals of *V. canescens* were used for the preparation of ligands. The three-dimensional structure of each chemical was retrieved from the PubChem database as an sdf file. With the aid of BIOVIA Discovery Studio Visualizer (version 21.1.0.20298), the files were transformed into pdb files. Finally, using the AutoDock program, chemicals were transformed into a pdbqt file [12].

#### 4.4.2. Proteins Selection and Preparation

The crystal structure of *Mus musculus* μOR bound to the activator BU72 was obtained from the Protein Data Bank (PDB reference Id: 5C1M). The structure of the *M. musculus* δOR bound to the opioid agonist naltrindole was solved and uploaded to the PDB with the accession number 4EJ4. Following the instructions, we downloaded enzymes and prepared them for docking investigations using BIOVIA Discovery Studio Visualizer (Dassault Systems: https://www.3ds.com, accessed on 5 January 2023) following Qasaymeh, et al. [30]. Polar hydrogens were added while water molecules and hetero atoms were removed.

#### 4.4.3. Active Site Prediction

The active sites of 5CIM and 4EJ4 were predicted through CASTp online server (http://cast.engr.uic.edu, accessed on 13 January 2023) and also from the literature. A protein structure’s pockets and voids are all identified by CASTp, which also provides a thorough breakdown of all the atoms involved in their production. Moreover, it analytically estimates the volume and area of each pocket and void by using both the molecular surface model (Connolly’s surface) and the solvent-accessible surface model (Richards’ surface) [31]. Amino acid residues shared by both proteins were chosen for this study. In order to ensure that the grid box set in the AutoDock Vina program encompasses the target protein’s binding site, the properly predicted amino acid residue must be used.

#### 4.4.4. Molecular Docking Analysis

Molecular docking of phytochemicals was performed using AutoDock Vina on two chosen protein targets of *M. musculus* [32]. Auto grid was used to cover the position of the ligand on the protein’s binding site using grid coordinates (X, Y, and Z axes) with default values and a grid spacing of 0.375. The output binding energies (B.E.) and dissociation constant (Kd) values, as well as ten iterations of the Lamarckian genetic algorithm (GA), were analyzed [33]. The BIOVIA Discovery Studio Visualizer was used to find the best docking orientations (those with the lowest binding energy and Kd value).

#### 4.4.5. Analysis of MD Simulation

The molecular activity and the protein–ligand complex’s stability were examined using MD simulations as they provide a broad perspective of the physical underpinnings of the investigated complex [34]. The current work analyzed the complex between the proposed protein 3D structures and ligands using the iMODS server [35]. This server’s benefit is speed with accurate estimates; it used standard mode analysis in internal coordinates to evaluate the collective motions of the complex [36].

#### 4.4.6. Physicochemical and Pharmacokinetic Properties of Phytocomponents

Absorption, distribution, metabolism, excretion, and toxicity are all abbreviated as ADMET in pharmacokinetics, pharmacology, and toxicology. It sheds light on a compound’s physicochemical, drug-likeness, and physiochemical characteristics. The idea behind ADMET is that a substance must be bioavailable, delivered to specific target locations, metabolized appropriately, eliminated after performing its intended function, and not have any adverse effects on the bodily cells, tissues, or organs with which it comes into contact. The drug discovery process is initiated with the prediction of a natural compound’s ADMET characteristics, a crucial step to determine the successful likelihood of clinical trials in general [37,38].

The identified compounds in the simplified molecular-input line-entry system (SMILES) format were exported to Swiss ADME and pkCSM web servers once the docking simulation was finished so they could be used in toxicity and bioavailability prediction processes such as Lipinski’s rule of five. Swiss ADME and pkCSM are free online resources for predicting small compounds’ pharmacokinetics, drug-likeness, and medicinal chemistry friendliness (http://www.swissadme.ch/ and http://biosig.unimelb.edu.au/pkcsm/prediction, Accessed on 21 January 2023) [9,39].

## 5. Conclusions

The findings of the current study suggest that secondary pharmacologically active metabolites found in a methanol extract of *V. canescens* may contribute to an explanation for the antidiarrheal action exhibited by the plant when tested in vivo. In castor oil-induced diarrhea assay, all extracts and fractions generally had antidiarrheal properties comparable to those of loperamide. In addition, the mobility of charcoal meal in the gastrointestinal tract was reduced by all extracts/fractions at selected dosages. According to our in vitro molecular docking results, emetine, quercetin, and violanthin could be potential agents for developing novel antidiarrheal drugs. Notably, quercetin exhibited the most potent inhibitory effect on the δOR 4EJ4 receptor, while emetine had the most potent inhibitory effect on the μOR 5C1M receptor. The assessment for drug-likeness and pharmacokinetic properties further prove their promising characteristics. Therefore, these chemicals deserve extensive biological investigations and clinical studies to elucidate the possible molecular mechanism and future therapeutic development.

## Figures and Tables

**Table 1:** The antidiarrheal potential of *V. canescens* in castor oil-induced assay.

Treatments	Dose (mg/Kg (b.w))	On Set of Diarrheal Symptoms (min) (Mean ± Std)	No. of Wet Feces (Mean ± Std)	% Antidiarrheal Activity (% Inhibition)	Weight of Wet Feces (Mean ± Std)	% Fecal Output
CME	100	105.2 ± 2.77 *	4.8 ± 0.84 *	48.94	3.64 ± 0.23 *	61.07
	200	114.2 ± 1.64 *	4.2 ± 0.45 *	55.32	3.29 ± 0.32 *	55.20
	300	135.2 ± 3.27 *	3.6 ± 0.55 *	61.70	2.97 ± 0.30 *	49.83
EAF	100	123.0 ± 2.55 *	4.4 ± 0.55 *	53.19	3.23 ± 0.15 *	54.19
	200	127.6 ± 2.41 *	3.4 ± 0.55 *	63.83	2.88 ± 0.07 *	48.32
	300	137.6 ± 1.14 *	3.2 ± 0.45 *	65.96	2.74 ± 0.05 *	45.97
AqF	100	071.4 ± 2.41 *	5.8 ± 0.84 *	38.30	3.97 ± 0.07 *	66.61
	200	079.8 ± 2.05 *	5.4 ± 0.55 *	42.55	4.07 ± 0.07 *	68.29
	300	085.4 ± 2.88 *	5.6 ± 0.55 *	40.43	4.25 ± 0.08 *	71.31
NHF	100	067.2 ± 2.28 *	6.4 ± 0.55 *	31.91	5.91 ± 0.26 *	99.16
	200	072.2 ± 3.77 *	6.0 ± 0.71 *	36.17	5.68 ± 0.06 *	95.30
	300	082.2 ± 1.64 *	5.4 ± 0.55 *	42.55	4.90 ± 0.23 *	82.21
ChF	100	133.4 ± 1.95 *	4.4 ± 0.55 *	53.19	3.36 ± 0.00 *	56.38
	200	143.4 ± 2.07 *	4.2 ± 0.45 *	55.32	3.33 ± 0.05 *	55.87
	300	149.4 ± 2.19 *	3.4 ± 0.55 *	63.83	3.04 ± 0.07 *	51.01
UCC	100	155.8 ± 2.59 *	4.6 ± 0.89 *	51.06	4.08 ± 0.28 *	68.46
	200	162.6 ± 1.14 *	4.0 ± 0.71 *	57.45	3.32 ± 0.20 *	55.70
	300	171.8 ± 1.64 *	3.4 ± 0.55 *	63.83	3.14 ± 0.06 *	52.68
CTA	100	157.6 ± 2.51 *	4.2 ± 0.45 *	55.32	3.39 ± 0.05 *	56.88
	200	167.4 ± 1.82 *	3.4 ± 0.55 *	63.83	3.12 ± 0.20 *	52.35
	300	184.0 ± 2.24 *	3.4 ± 0.55 *	63.83	2.92 ± 0.13 *	48.99
CTF	100	153.2 ± 1.30 *	4.6 ± 0.89 *	51.06	3.30 ± 0.21 *	55.37
	200	154.6 ± 2.61 *	4.0 ± 0.71 *	57.45	2.97 ± 0.17 *	49.83
	300	160.2 ± 2.17 *	4.2 ± 0.84 *	55.32	3.17 ± 0.05 *	53.19
Normal saline		061.8 ± 1.92	9.4 ± 1.14	--------	5.96 ± 0.45	-------
Loperamide		200.6 ± 2.07 *	1.6 ± 0.55 *	82.98	0.91 ± 0.06 *	15.27

* = significantly different from negative control (normal saline) at *p* < 0.01. std = standard deviation.

**Table 2:** The antidiarrheal potential of *V. canescens* in the charcoal meal assay.

Treatments	Dose	Total Length of Small Intestine (Mean ± Std)	Distance Traveled by Charcoal Means Mean ± Std)	Peristalsis Index	% Inhibition
Saline	10 mL/kg	54.42 ± 0.21	48.52 ± 0.97	89.16	00.00
CME	100 mg/kg	54.92 ± 0.32	36.98 ± 0.98 *	67.33 *	23.78
	200 mg/kg	53.65 ± 1.01	30.85 ± 0.54 *	57.50 *	36.42
	300 mg/kg	53.89 ± 0.12	31.63 ± 0.76 *	58.69 *	34.81
EAF	100 mg/kg	52.49 ± 0.43	36.34 ± 0.98 *	69.23 *	25.10
	200 mg/kg	51.37 ± 0.21	33.23 ± 0.34 *	64.69 *	31.51
	300 mg/kg	53.76 ± 2.32	29.99 ± 0.45 *	55.78 *	38.19
AqF	100 mg/kg	49.87 ± 0.23	39.82 ± 0.76 *	79.85 *	17.93
	200 mg/kg	53.76 ± 2.32	36.90 ± 0.82 *	68.64 *	23.95
	300 mg/kg	52.64 ± 1.27	34.09 ± 0.45 *	64.76 *	29.74
NHF	100 mg/kg	49.56 ± 2.01	35.79 ± 0.92 *	72.22 *	26.24
	200 mg/kg	48.09 ± 1.82	34.09 ± 1.02 *	70.89 *	29.74
	300 mg/kg	48.21 ± 1.23	33.54 ± 1.10 *	69.57 *	30.87
ChF	100 mg/kg	47.03 ± 1.34	26.06 ± 2.12 *	55.41 *	46.29
	200 mg/kg	47.98 ± 1.02	26.03 ± 0.95 *	54.25 *	46.35
	300 mg/kg	47.9 ± 2.120	23.04 ± 1.26 *	48.10 *	52.51
UCC	100 mg/kg	51.98 ± 2.10	34.09 ± 1.54 *	65.58 *	29.74
	200 mg/kg	48.92 ± 0.98	29.54 ± 1.73 *	60.38 *	39.12
	300 mg/kg	48.97 ± 0.87	25.98 ± 1.02 *	53.05 *	46.46
CTA	100 mg/kg	47.21 ± 2.16	25.86 ± 0.34 *	54.78 *	46.70
	200 mg/kg	51.02 ± 2.65	27.01 ± 0.56 *	52.94 *	44.33
	300 mg/kg	50.25 ± 2.19	24.98 ± 1.65 *	49.71 *	48.52
CTF	100 mg/kg	51.02 ± 0.98	29.02 ± 0.09 *	56.88 *	40.19
	200 mg/kg	50.21 ± 0.84	23.08 ± 0.64 *	45.97 *	52.43
	300 mg/kg	48.07 ± 0.94	20.34 ± 0.54 *	42.31 *	58.08
Atropine Sulfate	5 mg/kg	48.98 ± 1.21	16.32 ± 0.54 *	33.32 *	66.36

* = significantly different from negative control (normal saline) at *p* < 0.01. std = standard deviation.

**Table 3:** The docking scores of compounds against mu and delta-opioid receptors (μOR and δOR).

Source	Compound Name	Phytochemical Type	PubChem CID	Docking Score (kcal/mol) against Mu-Opioid Receptor (5C1M)	Docking Score (kcal/mol) against Delta-Opioid Receptor (4EJ4)
*Viola canescens*	Emetine	Alkaloid	10219	−9.7	−7.1
	Quercetin	Flavonoid	5280343	−8.1	−8.5
	Violanthin	Flavone	442665	−9.5	−7.9

**Table 4:** Drug-likeness and physiochemical properties of *Viola canescens* compounds through Swiss ADMET tool.

Compound No.	Compounds	Molecular Weight	Number of Hydrogen Bond Acceptors	Number of Hydrogen Bond Donor	Number of Rotatable Bonds	Mol. PSA
1	Emetine	480.64 g/mol	6	1	7	52.19
2	Quercetin	302.24 g/mol	7	5	1	131.36
3	Violanthin	563.48 g/mol	14	10	4	250.97

**Table 5:** The pharmacokinetic profile and toxicity prediction of *Viola canescens* compounds through pkCSM webserver.

ADMET Parameters	Emetine	Quercetin	Violanthin
Absorption			
Water solubility (log mol/L)	−3.666	−2.925	−2.894
Caco-2 permeability (log Papp, cm/s)	0.751	−0.229	−1.031
HIA (%)	91.032	77.207	31.604
Skin permeability (log Kp)	−2.798	−2.735	−2.735
Distribution			
VDss (human) (log L/kg)	1.596	1.559	1.227
BBB permeability (log BB)	−0.394	−1.098	−1.858
Metabolism			
CYP2D6	Yes	No	No
CYP3A4	Yes	No	No
Excretion			
Total clearance	0.993	0.407	0.073
Renal OCT2 substrate	No	No	No
Toxicity			
Ames test	No	No	No
Hepatotoxicity	No	No	No
Oral rat acute toxicity (LD50, in mol/kg	2.793	2.471	2.524

## Data Availability

Not applicable.
